# The overexpression of RBM3 alleviates TBI‐induced behaviour impairment and AD‐like tauopathy in mice

**DOI:** 10.1111/jcmm.15555

**Published:** 2020-07-10

**Authors:** Bingjin Liu, Yun Cao, Fangxiao Shi, Lin Wang, Na Li, Xiangshu Cheng, Jin Du, Qing Tian, Xinwen Zhou

**Affiliations:** ^1^ Department of Pathophysiology Key Laboratory of Neurological Diseases of Education Ministry Tongji Medical College Huazhong University of Science and Technology Wuhan China; ^2^ School of Medicine and Pharmaceutical Engineering Taizhou Vocational and Technical College Taizhou China; ^3^ Department of Neurology Center for Translational Medicine Huaihe Hospital of Henan University Kaifeng China

**Keywords:** cognitive deficits, hypothermia pre‐treatment, RNA‐binding motif protein 3, tau phosphorylation, traumatic brain injury

## Abstract

The therapeutic hypothermia is an effective tool for TBI‐associated brain impairment, but its side effects limit in clinical routine use. Hypothermia up‐regulates RNA‐binding motif protein 3 (RBM3), which is verified to protect synaptic plasticity. Here, we found that cognitive and LTP deficits, loss of spines, AD‐like tau pathologies are displayed one month after TBI in mice. In contrast, the deficits of LTP and cognitive, loss of spines and tau abnormal phosphorylation at several sites are obviously reversed in TBI mice combined with hypothermia pre‐treatment (HT). But, the neuroprotective role of HT disappears in TBI mouse models under condition of blocking RBM3 expression with RBM3 shRNA. In other hand, overexpressing RBM3 by AAV‐RBM3 plasmid can mimic HT‐like neuroprotection against TBI‐induced chronic brain injuries, such as improving LTP and cognitive, loss of spines and tau hyperphosphorylation in TBI mouse models. Taken together, hypothermia pre‐treatment reverses TBI‐induced chronic AD‐like pathology and behaviour deficits in RBM3 expression dependent manner, RBM3 may be a potential target for neurodegeneration diseases including Alzheimer disease.

## 1 INTRODUCTION

Alzheimer disease (AD) is the most common form of dementia in older individuals and probably develops as a result of complex interactions among multiple factors, including age, genetics, environment, lifestyle and traumatic brain injury (TBI).^1^, ^2^, ^3^, ^4^, ^5^ The evidence has shown that there appears to be a strong link between future risk of Alzheimer's and serious TBI, but how TBI increase future risk of AD is still unclear.^6^, ^7^, ^8^

TBI caused by contact sports, work accident or military blasts may induce acute or potentially long‐lasting neurological dysfunction, including chronic traumatic encephalopathy.^4^, ^5^, ^6^, ^8^, ^9^ The clinical features of TBI vary depending on the mechanistic types and severity of head injury. These features generally consist of cognitive symptoms such as deficits in memory and capacity of learning new information. The cumulative evidence^7^, ^10^, ^11^ has shown that TBI can induce AD‐like tau hyperphosphorylation, extensive dendrite and spine degeneration as well as a significant reduction in the number of synapses in rodents, which has been as a model of AD for exploring inherent relationship between TBI and AD.

While the potential beneficial effects of hypothermia (HT) in the treatment of TBI have been reported in the literature since the mid‐1940s,^12^ the clinical practice has observed that post‐traumatic hypothermia is an effective technique for reducing cerebral metabolism and oxygen consumption, diminishing cytotoxic oedema, stabilizing the blood‐brain barrier.^13^ Furthermore, hypothermia has often been shown to improve survival and outcome in animal models of TBI.^14^, ^15^ The side effects of post‐traumatic hypothermia, such as increased risk of infection, cold diuresis and hypovolaemia, electrolyte disorders, insulin resistance, impaired drug clearance and mild coagulopathy, limit the extensive use, so that clinical benefits of HT in TBI are still under debate.^16^ Therefore, the extensive use of hypothermia in TBI treatment needs to avoid its side effects and exert its brain protection as much as possible. A large number of studies have proved that TBI can induce AD‐like tau pathological damage and brain dysfunction in addition to acute brain injuries while post‐traumatic hypothermia can significantly improve the brain function of TBI mice.^17^, ^18^, ^19^, ^20^, ^21^ For alleviating side effects of post‐traumatic hypothermia and exploring a tool to prevent TBI‐mediated neurodegeneration, we wanted to determine which molecular induces the neuroprotection of HT in TBI mice.

The clinical practice has shown that therapeutic hypothermia (32‐34°C) is a potent tool to alleviate neurological deficits in infants and in adults caused by hypoxic‐ischemic encephalopathy^21^, ^22^ or acute brain injuries.^13^, ^23^ RBM3 protein syntheses peak in a range of mild to moderate temperatures,^24^ while there is lesser life‐threatening side effects in the hypothermia (32‐34°C) treatment than in that of deeper hypothermia treatment, such as cardiac and transplant surgery. RNA‐binding motif protein 3 (RBM3), a member of the glycine‐rich RNA‐binding protein (GRP) family, is one of the first proteins synthesized in response to hypothermia and hypoxia.^25^, ^26^, ^27^, ^28^ Although many studies on RBM3 have been focused on their regulation in non‐neuronal cells in response to hypothermia and other stress factors,^29^, ^30^ there is growing interest in their role as effectors in therapeutic hypothermia‐induced neuroprotection.^24^, ^31^, ^32^ Furthermore, RBM3 has been shown to play a major role in promoting translation in neuronal cells,^33^ and recently, RBM3 up‐regulation in neuronal cells in response to hypothermia has been implicated in hypothermia‐induced neuroprotection,^24^, ^31^, ^34^ suggesting that RBM3 may involve in the protective effects of HT.

To elucidate the role and mechanism of RBM3 in protecting brain from TBI‐caused chronic injury benefits us understanding the neuroprotective effects of hypothermia and the biological function of RBM3. Therefore, we speculated that RBM3 plays an important role in the neuroprotective effect of hypothermia pre‐treatment against TBI‐induced chronic impairments. In this study, we found that hypothermia pre‐treatment could induce expression of RBM3, meanwhile the TBI‐induced spatial learning and memory deficits and the loss of spines, LTP inhibition and AD‐like tau phosphorylation are also reversed completely or partly, as well as that the protective effect of hypothermia pre‐treatment on the brain of TBI mice is abolished when blocking expression of RBM3. Whatever, increasing expression of RBM3 could mimic neuroprotective role of HT. Taken together, up‐regulating RBM3 expression plays a crucial role in hypothermia pre‐treatment attenuating TBI‐induced chronic AD‐like pathology and behaviour deficits.

## 2 METHODS AND MATERIALS

### 2.1 Animals and treatment

Male C57BL/6 mice (3 months old, weight 28 ± 2 g) were used for experiments. All animals were housed under a 12‐h/12‐h day/night cycle and kept free access food and water. The animal experiments were approved by the Ethics Committee of Tongji Medical College and performed according to the ‘Policies on the Use of Animals and Humans in Neuroscience Research’ by the Society for Neuroscience in 1995.

Mice were randomly divided into eight groups: Sham (Normal control), Sham + HT, TBI, TBI + AAV‐control, TBI + AAV‐RBM3, TBI + HT, TBI + HT+AAV‐ control and TBI + HT+shRNA‐RBM3. There were 12 to 15 mice in each group. The hypothermia treatment followed previous method.^34^ Briefly, mice were firstly injected intraperitoneally with 5’‐AMP (0.7 mg per g) or the same amount of saline and then were placed at room temperature for about 1 hour. When the body temperature dropped to 25°C, the mice infused by 5’‐AMP were transferred to 5°C freezer and the core body temperature of mice dropped to 16‐18°C for 45 minutes. The mice were maintained at room temperature to recover normal body temperature. Mice in the HT groups were cooled twice (once a week) two weeks before TBI treatment. TBI and sham‐injury procedures operated in accordance with previously described methods.^7^, ^35^ In short, mice were anaesthetized with 4% isoflurane in a 70:30 air‐oxygen mixture in a sealed Plexiglas box. The anaesthetized animals were positioned on the impact device, and the midpoint of the posterior fontanel and the anterior fontanel was placed directly below the hollow 60‐inch height guide tube. A 54‐g weight metal bolt was released from the upper mouth of the tube, causing an impact to the dorsal aspect of the skull and resulting in a rotational acceleration of the head.^8^ Mice were removed from the device after the impact and recovered in room air. Sham‐injured mice were anaesthetized but not impacted. After underwent HT treatment or adeno‐associated virus (AAV)‐RBM3 plasmid injection or HT + shRNA‐RBM3 or empty vector, mice were performed TBI or sham treatment.

### 2.2 Adeno‐associated virus injection

After anaesthesia, the anterior and posterior fontanels of mice were placed on the same level in the stereotactic positioning device and then disinfected with iodine wine. Two holes were drilled symmetrically 2.0 mm posterior to the bregma and 2.2 mm laterally from the midline, and deep 2.1 mm under the surface of the brain. The AAV‐RBM3 plasmid, shRNA‐RBM3 or empty vector (AAV‐control) of 1.5 μL, was injected into the hippocampal CA3 area, respectively. The injection speed was 0.2 μL/min and kept constant. After the injection, the needle was placed for 5 minutes, and the needle was screwed out slowly, and then, the meninges and skin were sutured. After the operation, the mice were placed in an empty cage, lying on their side and keeping the space between the mice until they recovered.

### 2.3 Morris water maze test

The Morris water maze test began 3 weeks after TBI or sham injury. The water maze consisted of a round tank filled with water (temperature at 22‐25°C). A hidden platform (10 cm in diameter) was placed at the fixed position of the third quadrant and submerged 1 cm under the water surface. For spatial learning, mice were trained to find the platform for 6 consecutive days (three trials per day). The training began from the first, second and fourth quadrant, respectively. In each trial, mice were given 60 seconds to search for the platform until they landed on it or were gently guided to it if they failed to find the platform within the 60 seconds. The mice were allowed to stay the platform for 30 seconds. For probe trial, the platform was removed on day 7. The escape latency time, the time spent in the target quadrant, the number of platform crossing and swimming speeds were monitored by a computerized tracking system.

### 2.4 Golgi staining

The anaesthetized mice were perfused with 0.9% saline containing 0.5% sodium nitrite, followed by 4% formaldehyde and then with a staining solution composed of 4% formaldehyde, 5% chloral hydrate and 5% potassium dichromate. The brains were removed and immersed in the same dye solution for 3 days and then transferred to 1% silver nitrate solution for another 3 days in the dark. The brains were sliced by the vibratome at a thickness of 40 μm. Golgi staining neuronal spine densities was randomly determined in segments of dendrites at a distance of 90 μm from the soma, counted in z‐stacks by manual scrolling of the images. The images were obtained under bright‐field microscopy (Axioplan 2; Zeiss, Brighton, MI, USA). The dendritic spines from at least 60 neurons per group were counted with Neurolucida software (MicroBrightField, Williston, VA, USA). Ten well‐impregnated neurons that were clearly distinguishable from others were analysed for each group. Spinal density was calculated per 10 μm of dendritic length. All tracings and analyses were performed in a blind manner.

### 2.5 Extracellular recordings in brain slice

Electrophysiological recordings were administered with standard procedures^36^ at the end of behavioural tests. Coronal slices (400 μm) were cut with the vibratome and incubated for 1 hour in a continuously flowing artificial cerebrospinal fluid (ACSF) (2‐3 mL/min, 30°C) gassed with 5% CO_2_ and 95% O_2_. The composition of the control solution was (in mM) as follows: 124 NaCl, 3.0 KCl, 26 NaHCO_3_, 1.0 MgCl_2_, 1.25 NaH_2_PO_4_, 2.0 CaCl_2_ and 10 glucose. Slices were transferred to a recording chamber submerged in ACSF and stimulated in CA3 using glass microelectrodes. fEPSPs were recorded in the stratum radiatum of the CA1. Stimulation intensity was adjusted to 40% of maximal size to evoke fEPSP amplitudes. LTP was induced by a set of high frequency stimulation (100 Hz, 1‐seconds duration at test strength).

### 2.6 Immunohistochemistry and Immunofluorescence

The mice were killed under anaesthetization and transcardially perfused with 200 mL normal saline and then with 400 mL 4% paraformaldehyde solution. The brains were removed and fixed in the same fixation fluid for another 24 hour at 4°C. For each primary antibody, five consecutive sections from each brain were used. The immunoreaction was detected with avidin horseradish peroxidase‐labelled antibodies and visualized with the diaminobenzidine tetrachloride system. The immunofluorescence was measured by the fluorescent secondary antibody (1:200, A‐11034 Invitrogen) for 1 hour. The images were observed with a microscope. For each experimental group, we measured 10 random images (6 slices from 3 mice). The Image‐Pro Plus 4.5 system (Media Cybernetics Inc, Rockville, USA) was used to estimate the intensity of the immunohistochemical reaction.^37^

### 2.7 Western blotting

The Western blotting was performed as described previously.^38^, ^39^ Briefly, the dissected tissues of hippocampal or cortex were homogenized in RIPA buffer and then centrifuged (5000× g) for 10 minutes. The supernatant was collected, and the protein levels were detected. The proteins in the extracts were separated by SDS/PAGE and then analysed by Western blotting using primary antibodies against RBM3 (1:500; Abcam (Cambridge, UK)), pT231 (1:1,000; SAB (Maryland, USA)), pS396 (1:1,000; Abcam), Tau5 (1:500; Millipore (Billerica, MA, USA)) and β‐actin (1:1,000; Abcam, ab6275), respectively. Membranes were incubated with Odyssey secondary antibody (1:10,000) at room temperature for 1 hour. Immunoreactive bands were visualized with the Odyssey Infrared Imaging System (Li‐COR; Biosciences, (Dublin, Ireland)) and quantitatively analysed with Image software.

### 2.8 Statistical analyses

The data were expressed as the mean ± SEM and analysed with SPSS 18.0 statistical software (SPSS Inc, Chicago, IL, USA). The one‐way analysis of variance or Student's *t* test was used to determine the different means among the groups. The level of significance was defined as *P* < .05.

## 3 RESULTS

### 3.1 Hypothermia pre‐treatment alleviates tau hyperphosphorylation one month after TBI in mice

**FIGURE 1:**
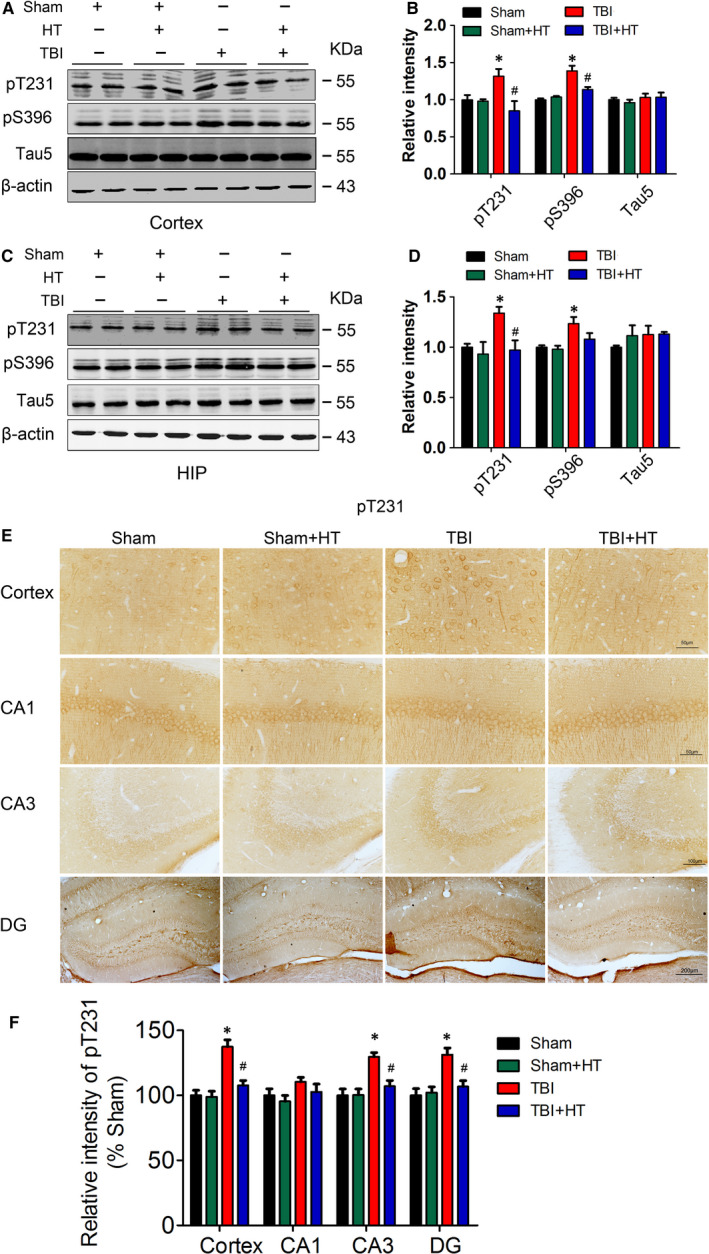
Hypothermia pre‐treatment reduces TBI‐induced AD‐like tau phosphorylation. The mice were performed hypothermia or sham treatment and then underwent TBI or sham. One mother after TBI or sham treatment, the hippocampal or cortex was homogenized in RIPA buffer and targets protein were measured by Western blotting. The expression of the pT231, pS396 and Tau5 in the extracts of cerebral cortex (A) or hippocampi (C) and quantitative analysis (B and D). The band used for quantification was specified at 55 Kda. The size of molecular weight in WB is marked in the revised manuscript. The five consecutive sections from each brain were stained with avidin horseradish peroxidase‐labelled antibodies and visualized with the diaminobenzidine tetrachloride system. The representative immunostaining image (E) and quantitative analysis (F). One‐way ANOVA. All data were expressed as the mean ± SEM (n = 10, male). *P < .05 vs. Sham; ^#^ *P* < .05 vs. TBI

In our previous study, we found hypothermia pre‐treatment could attenuate the impairment in LTP and cognitive function induced by TBI.^35^ TBI‐induced progressive neurodegeneration has been shown to be a tau protein‐related disorder,^6^, ^39^ and tau is prone to high phosphorylation in response to single or multiple neurological traumas. To determine whether the chronic effects of TBI on tau phosphorylation occurs, one month after TBI, we examined the phosphorylation of tau in the cerebral cortex and hippocampus of mice in our original animal model. The level of phosphorylated tau protein at T231, S396 sites in the cortex and hippocampus was obviously enhanced in TBI mice as compared with sham mice; meanwhile, hypothermia pre‐treatment significantly reduced TBI‐induced phosphorylation of tau protein at these sites in TBI + HT mice (Figure 1A‐D). The images of immunohistochemistry showed that the immunostaining of antibody for tau phosphorylation at T231 site was significantly increased in TBI mice as compared with sham mice in different region and hypothermia pre‐treatment also attenuated the immunostaining of phosphorylated tau antibody (Figure 1E‐F). The total amount of tau protein (Tau5) in each group was not significantly different (Figure 1A‐D). Taken together, we consider that TBI can induce chronic tau hyperphosphorylation and the tau hyperphosphorylation could be improved by hypothermia.

Dysfunction of learning and memory, hyperphosphorylation of tau protein and inhibition of LTP are the clinical or pathological features of AD patients.^40^ Therefore, the lesions caused by TBI are considered to be AD‐like lesions.

### 3.2 Hypothermia pre‐treatment induced increased expression of RBM3 protein

In clinical practice, therapeutic hypothermia (32‐34°C) has been proved a potent tool to alleviate neurological deficits in infants with hypoxic‐ischemic encephalopathy and in adults with acute brain injuries. Evidence has demonstrated that RBM3 plays an important neuroprotective role of hypothermia through preventing neuronal loss and restoring synapse reassembly in Alzheimer's and prion disease models.^34^ In addition, RBM3 induction is extremely sensitive to temperature change at least in neural cells in mouse.^34^, ^41^ To explore whether hypothermia pre‐treatment also induces expression of RBM3 protein and RBM3 is a crucial factor for hypothermia‐mediating neuroprotection. We detected the expression of RBM3 in the hippocampus of mice by Western blotting and immunohistochemistry.

**FIGURE 2:**
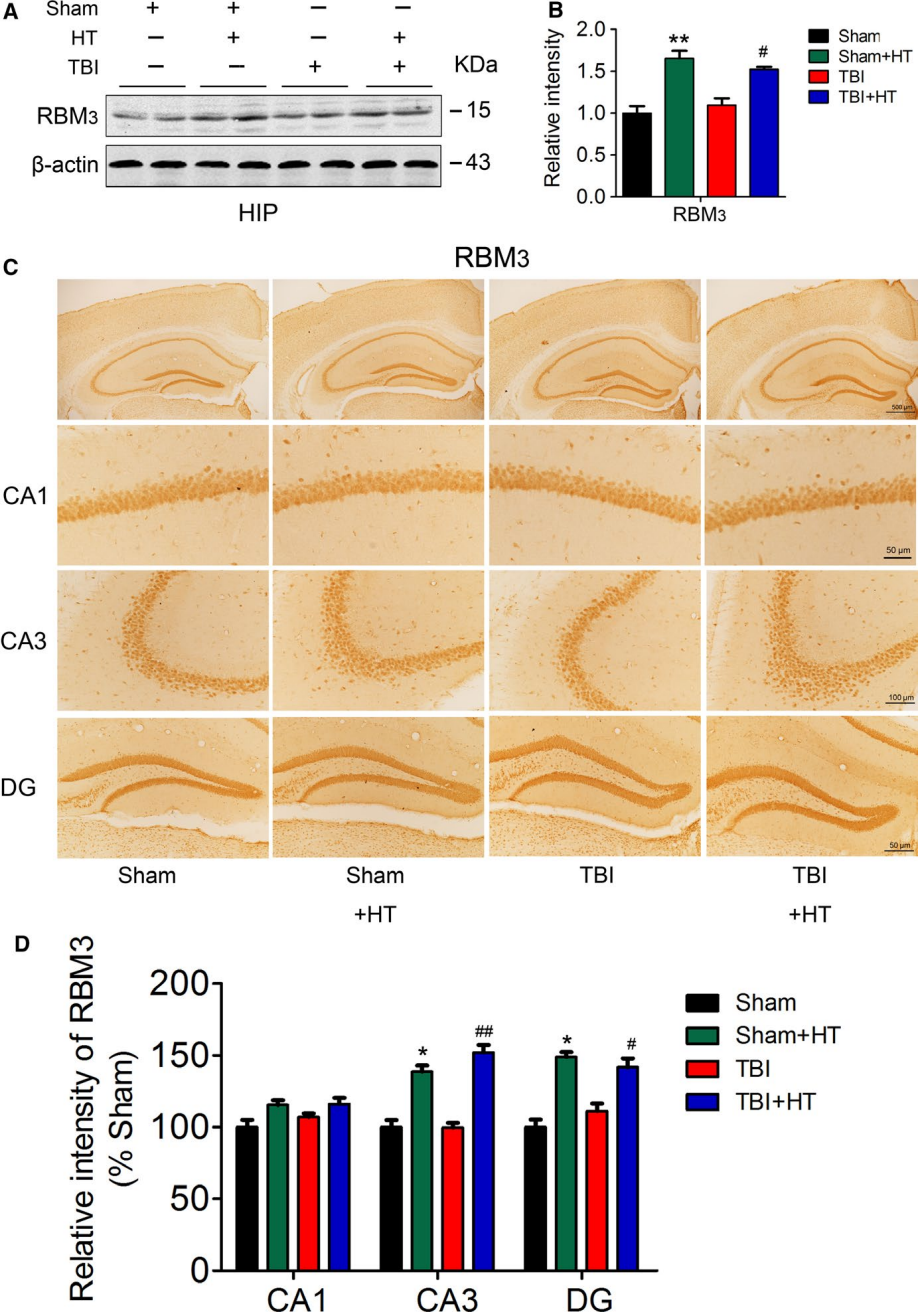
Hypothermia pre‐treatment induces RBM3 expression in mice. The expression of the RBM3 in the extracts of hippocampi of mice was detected by Western blotting (A) and quantitative analysis (B). The representative immunostaining image (C) 1 m after TBI or sham treatment and quantitative analysis (D). One‐way ANOVA. All data were expressed as the mean ± SEM (n = 10, male). **P < .01 vs. Sham; ^#^ *P* < .05 vs. TBI

The level of RBM3 was significantly elevated in the hippocampus in Sham + HT or TBI + HT group (about 1.5‐1.6 times), as compared with Sham or TBI group in one month after TBI (Figure 2A‐B). As shown as Figure S1A‐D, up‐regulation of RBM3 was more in 48h after TBI (two weeks after the first hypothermia) than in the cortex (about 1.7‐1.9 times) or hippocampus (about 1.9‐2.4 times) in one month after TBI. The immunostaining for RBM3 in the hippocampus was obviously stronger in Sham + HT or TBI + HT group than that of Sham or TBI group, which were consistent with the results of Western blotting (Figure 2C‐D), suggesting that hypothermia pre‐treatment significantly induced expression of RBM3 protein in normal and TBI mice.

### 3.3 RBM3 is required for HT improving TBI‐induced spatial learning and memory

**FIGURE 3:**
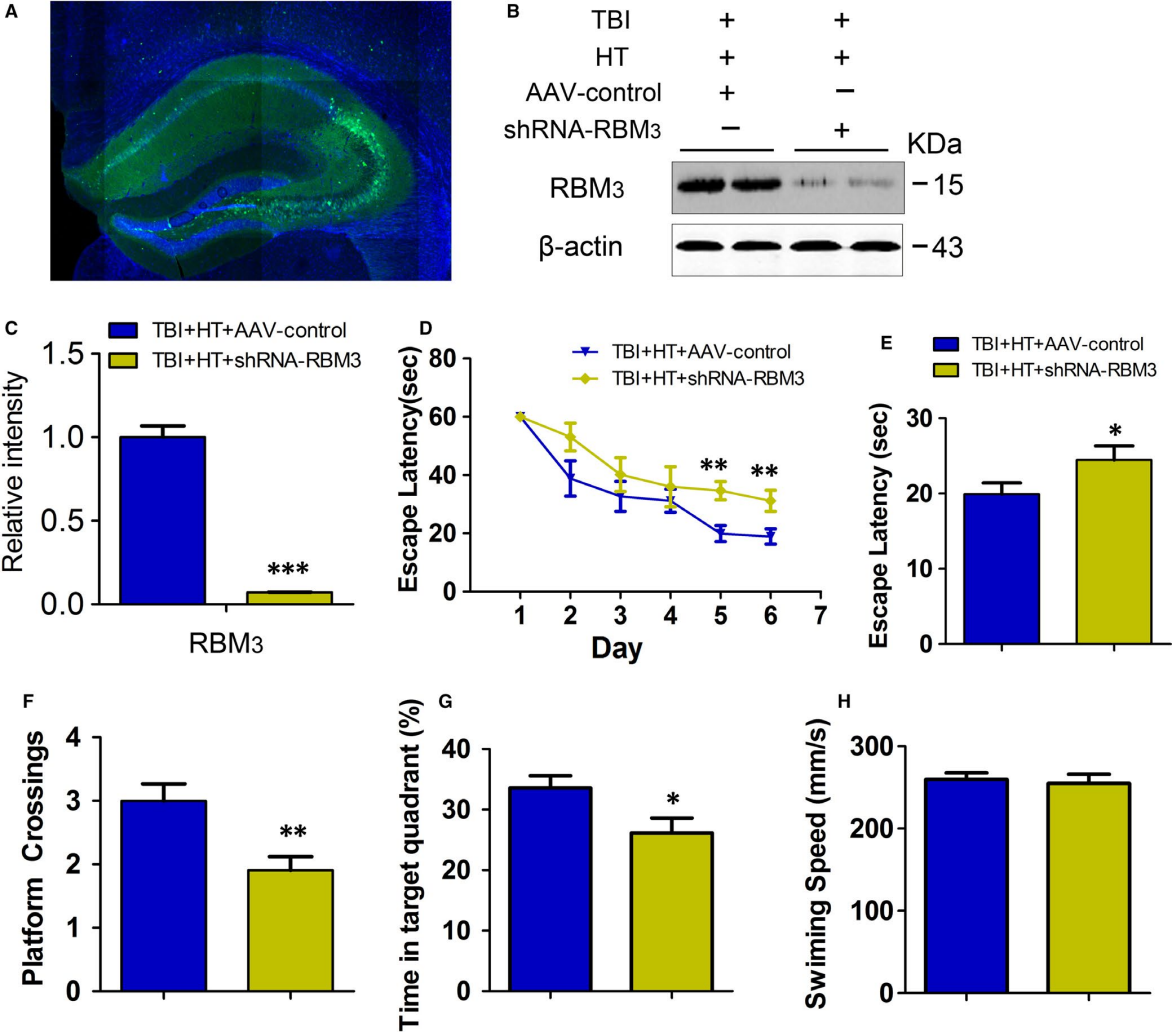
Blocking RBM3 expression reduces protection of hypothermia pre‐treatment against impairment of spatial learning and memory. The AAV‐shRNA‐RBM3 or empty vector (AAV‐control) of 1.5 μL was injected into the hippocampal CA3 area with microinjector, respectively, and then, the mice underwent a time sequence treatment including sham, hypothermia or TBI. The representative image of green fluorescence protein showed the plasmid expression of adeno‐associated virus carrying RBM3 or shRNA plasmid or empty plasmid (A). The expression of the RBM3 in the extracts of hippocampi (B) of mice was detected by Western blotting and quantitative analysis (C) 1 m after TBI or sham treatment. Mice were trained to find the platform for 6 consecutive days (three trials per day) and probe trial on day 7. The escape latency time (sec) (D), the latency to find the platform (E), the count of platform crossings (F), the time spent in the target quadrant (G), the average swimming speed during the probe test (H). Student's t test; two tailed. All data were expressed as the mean ± SEM (n = 10, male). *P < .05, **P < .01, ***P < .001 vs. TBI + HT+AAV‐control

The increased expression of RBM3 protein and decreased phosphorylation of tau co‐exist in TBI model treated by hypothermia. To further disclose whether RBM3 is required for the neuroprotective effect of hypothermia pre‐treatment in TBI mice, we block the expression of RMB3 in hippocampus by RBM3 shRNA plasmid in TBI + HT mice. The number of dendritic spines of neurons in the cortex and hippocampus decreased significantly after TBI in this experiment, especially the CA3 region of the hippocampus. So, we injected the adeno‐associated virus into the CA3 region. The adeno‐associated virus (AAV) carrying RBM3 shRNA plasmid or empty plasmid was fused into CA3 region of hippocampus in mice two weeks before hypothermia in TBI mice. The image of green fluorescence showed that the adeno‐associated virus carrying RBM3 shRNA plasmid or empty plasmid was delivered and expressed in CA3 (Figure 3A).Compared with TBI + HT + AAV‐control group, the level of RBM3 in TBI + HT + shRNA‐RBM3 group was significantly reduced in AAV injection region, suggesting that shRNA‐RBM3 effectively blocks HT‐induced RBM3 expression in TBI mice (Figure 3B‐C).

The previous and current studies have demonstrated that hypothermia reverses TBI‐caused AD‐like tau phosphorylation and brain dysfunction including impairment of LTP and spatial learning and memory.^7^, ^42^ The ability of spatial learning and memory of all experimental mice is evaluated by Morris water maze. The escape latencies on day 5, 6 in the orientation navigation experiment were longer in the TBI + HT + shRNA‐RBM3 than in TBI + HT + AAV‐control. The time of escape latency of TBI + HT + shRNA‐RBM3 mice on day 7 in the spatial probe test was significantly increased compared to that of TBI + HT + AAV‐control mice, and the time of escape latency of TBI + HT + shRNA‐RBM3 was obviously longer that of TBI + HT + AAV‐control. The change tendency of crossing platform times and time in the target quadrant was completely same as the time of escape latency and the swimming speed was no change in different group (Figure 3D‐H). These data of behaviour suggested that hypothermia improves TBI‐caused brain function impairment in RBM3‐dependent manner.

### 3.4 HT improves impairment of spine and LTP caused by TBI in RBM3‐dependent manner

**FIGURE 4:**
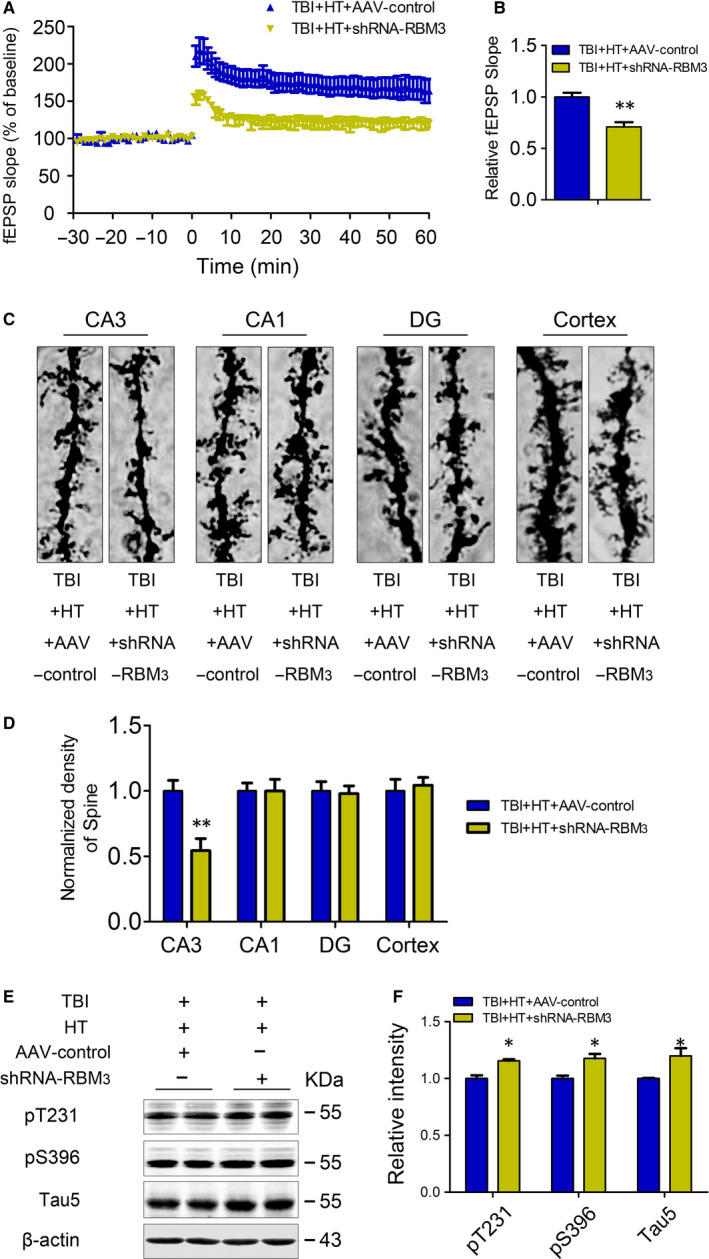
HT improves TBI‐induced impairment of LTP and spine caused by TBI via RBM3. The coronal slices (400 μm) were cut with the vibratome and incubated for 1 h in a continuously flowing artificial cerebrospinal fluid (ACSF). Slices were transferred to a recording chamber submerged in ACSF and stimulated in CA3 using glass microelectrodes, and fEPSPs were recorded in the stratum radiatum of the CA1. The slope of fEPSP after HFS is normalized by the baseline. The onset of HFS at 0 min; the traces are average fEPSPs from five sweeps before (−30 min‐0 min) and after (0 min‐60 min) LTP induction (A). Quantitative analyses for fEPSPs measured 40‐60 min after HFS relative to baseline (B). The brains stained by silver nitrate were sliced by the vibratome at a thickness of 40 μm. Golgi staining neuronal spine densities were randomly determined in segments of dendrites at a distance of 90 μm from the soma and counted in z‐stacks by manual scrolling of the images. The images were obtained under bright‐field microscopy (Axioplan 2; Zeiss, Brighton, MI, USA). The dendritic spines from at least 60 neurons per group were counted with Neurolucida software (MicroBrightField, Williston, VA, USA). All tracings and analyses were performed in a blind manner. The representative image of spine density in the hippocampus was measured via Golgi stain (C). Quantitative analysis of the spine numbers (at least 15 neurons, three dendritic branches per neuron, from three mice, were used for the analysis) (D). The expression of the pT231, pS396 and Tau5 in the extracts of hippocampi of mice was detected by Western blotting (E) and quantitative analysis (F) 1 m after TBI or sham treatment. Student's t test; two tailed. All data were expressed as the mean ± SEM, (n = 10, male). *P < .05, **P < .01 vs. TBI + HT+AAV‐control

The previous studies have demonstrated that the capacity of spatial learning and memory is associated with synapse plasticity and long‐term potential in mice.^43^ The field excitatory postsynaptic potentials (fEPSP) were recorded in the CA1 region in response to stimulation inputs from CA3 in brain slices. Our previous results showed that the LTP is impaired in TBI mice, and hypothermia could effectively improve the impaired LTP by TBI in mice. Here, we found that the impairment of LTP occurred again when blocking RBM3 expression under HT + TBI conditions (Figure 4A‐B). Evidence has demonstrated that the synapse plasticity can be evaluated using number of spines.^44^ To observe the changes in dendrites in different administration mice, the Golgi stain was performed at the end of the behaviour test. Golgi stain data showed that the dendritic spine number in CA3 area of hippocampus in TBI + HT + shRNA‐RBM3 mice was significantly decreased when blocking RBM3 expression in CA3 area, while no significant difference was found in other brain regions (Figure 4C‐D).

### 3.5 Blocked expression of RBM3 eliminates the neuroprotective role of hypothermia pre‐treatment for TBI‐induced AD‐like tau phosphorylation

The increased expression of RBM3 protein and decreased phosphorylation of tau co‐exist in TBI model treated by hypothermia. We speculated that hypothermia attenuates TBI‐induced AD‐like tau pathology through RBM3. So, we blocked the expression of RMB3 in CA3 region of hippocampus by RBM3 shRNA plasmid in TBI + HT mice. Compared with TBI + HT + AAV‐control group, the decreased p‐tau at pT231, pS396 sites are restored in TBI + HT + shRNA‐RBM3 group (Figure 4E‐F), suggesting that the role of hypothermia pre‐treatment reducing TBI‐induced tau hyperphosphorylation is also dependent RBM3 expression.

### 3.6 Overexpression of RBM3 reduces AD‐like lesions in mice

**FIGURE 5:**
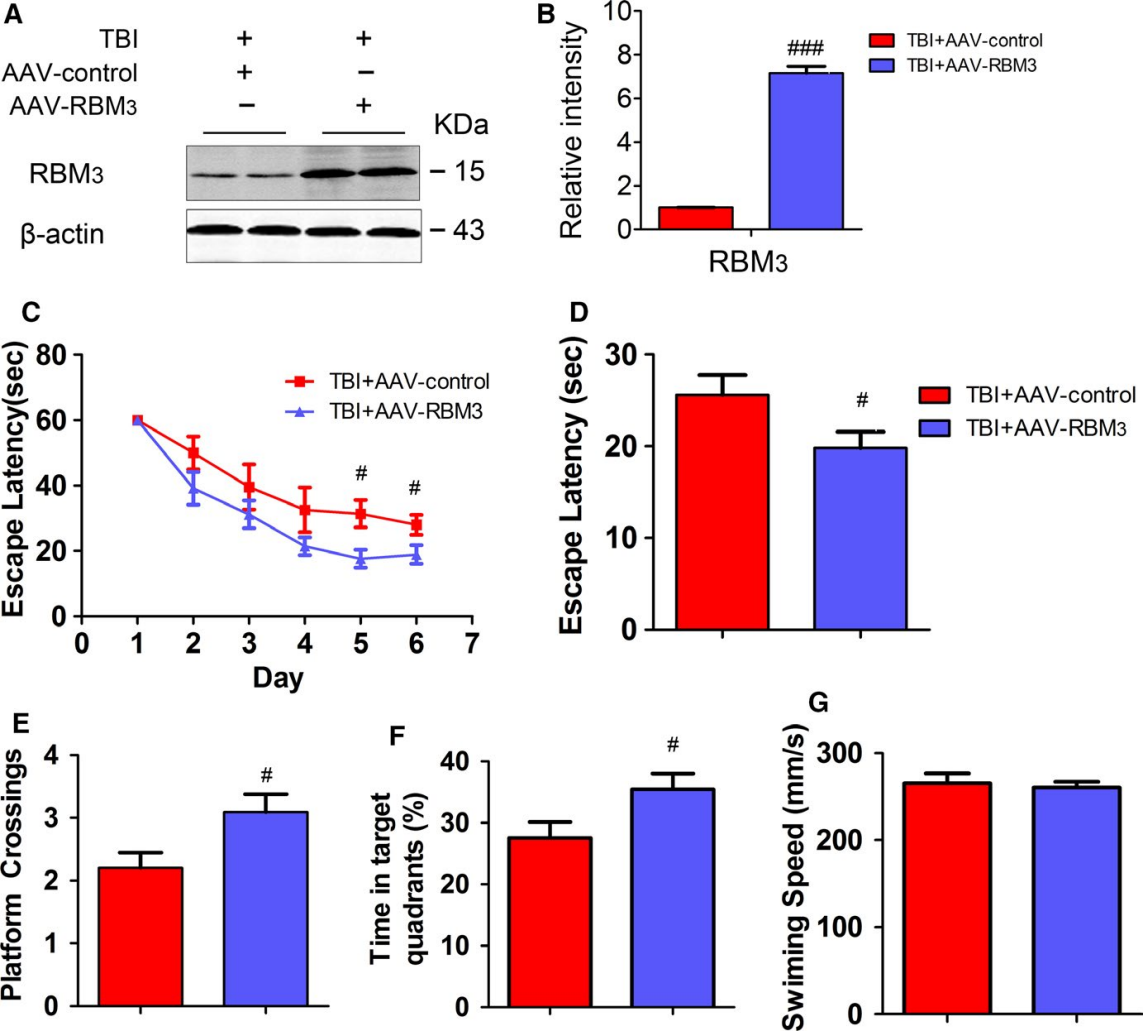
Overexpression of RBM3 restores capacity of learning and memory of TBI mice. The AAV‐RBM3 or empty vector (AAV‐control) of 1.5 μl was injected into the hippocampal CA3 area with microinjector, respectively, and then, the mice underwent a time sequence treatment including sham or TBI. The expression of the RBM3 in the extracts of hippocampi of mice was detected by Western blotting (A) and quantitative analysis (B) 1 m after treatment. Mice were trained to find the platform for 6 consecutive days (three trials per day) and probe trial on day 7. The escape latency time (sec) (C), the latency to find the platform (D), the count of platform crossings (E), the time spent in the target quadrant (F), the average swimming speed (G) during the probe test. Student's t test; two tailed. All data were expressed as the mean ± SEM (n = 10, male). ^#^ *P* < .05, ^###^ *P* < .001 vs. TBI + AAV‐control

**FIGURE 6:**
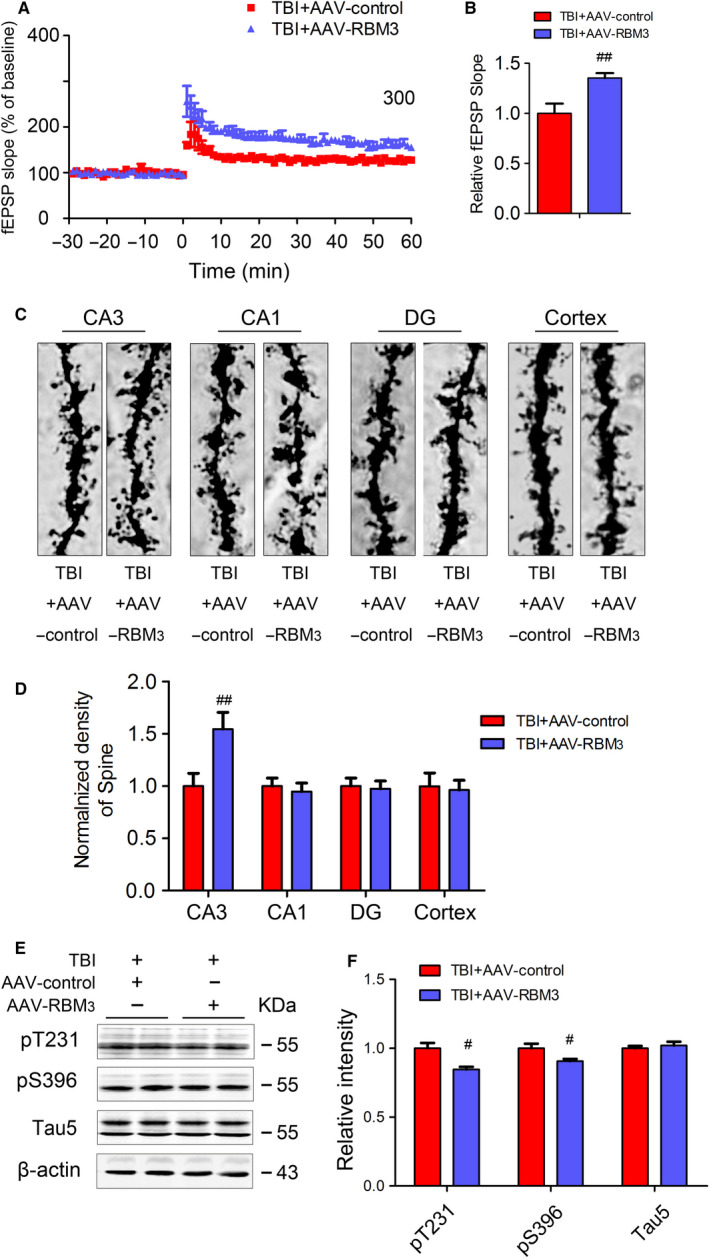
Overexpression of RBM3 improves TBI‐induced impairment of LTP and spine, and tau hyperphosphorylation. The traces are average fEPSPs from five sweeps before (−30 min‐0 min) and after (0 min‐60 min) LTP induction (A). Quantitative analyses for fEPSPs measured 40‐60 min after HFS relative to baseline (B). Golgi staining neuronal spine densities were randomly determined in segments of dendrites at a distance of 90 μm from the soma and counted in z‐stacks by manual scrolling of the images. The representative image of spine density in the hippocampus was measured via Golgi stain (C). Quantitative analysis of the spine numbers (D). The expression of the pT231, pS396 and Tau5 in the extracts of hippocampi of mice was detected by Western blotting (E) and quantitative analysis (F) 1 m after TBI or sham treatment. Student's t test; two tailed. All data were expressed as the mean ± SEM, (n = 10, male). ^#^ *P* < .05, ^##^ *P* < .01 vs. TBI + HT + AAV‐control

To further determine role of RBM3 in improving TBI‐induced AD‐like lesions, the AAV‐RBM3 was injected into CA3 of hippocampus in mice using stereotaxic apparatus. The expression of RBM3 obviously increases in TBI + AAV‐RBM3 group compared to TBI + AAV‐control group in 8 weeks after AAV‐RBM3 administration (Figure 5A‐B). In contrast with blocking RBM3 expression under HT condition, we found that the time of escape latency in TBI + AAV‐RBM3 mice is obviously shorter than that of TBI + AAV‐control mice and crossing platform times and time in the target quadrant showed the same change tendency as the time of escape latency (Figure 5C‐G); the LTP impairment by TBI also is restored when overexpressing RBM3 (Figure 6A‐B) and overexpression RBM3 in CA3 area could restored the decreased the dendritic spine number caused by TBI (Figure 6C‐D), suggesting that overexpression of RBM3 also improves TBI‐mediated impairment of spatial learning and memory and synaptic plasticity in mice. In addition, the levels of enhanced tau phosphorylation at pT231, pS396 sites in TBI + AAV‐RBM3 group are reversed compared with TBI‐AAV‐control group (Figure 6E‐F), suggesting that overexpression of RBM3 also reduces TBI‐induced tau phosphorylation in mice, which mimics HT‐like neuroprotective role. Taken together, the current data suggested that HT restores capacity of learning and memory of TBI mice in RBM3‐dependent manner.

## 4 DISCUSSION

The traumatic brain injury (TBI) results from direct or indirect exposure to an explosive event as may occur in domestic or industrial accidents, sports contact, terrorist attacks or in military conflict, which leads to prolonged cognitive, sensory, personality disorders and even death in human and animals. Growing evidence also has shown that TBI also causes chronic encephalopathy and increases the risk of neurodegeneration diseases in future, such as Alzheimer disease. The previous studies have demonstrated that TBI can induce AD‐like pathologies and cognitive deficits in rodents. In the current study, we established as an AD‐like mouse models by TBI. The TBI mice display AD‐like learning and memory impairments, AD‐like tau phosphorylation at several sites and loss of synapses one month after TBI, which is consistent with previous studies.^7^, ^34^ These data support an opinion that TBI and AD share similar pathological features such as the presence of abnormal tau phosphorylation (main component of neurofibrillary tangles), loss of synapse and cognitive deficits.^45^ As we known, there are currently no efficient drugs for AD. However, the shared pathology may indicate that TBI mouse models can be used to explore the mechanisms and therapy of AD.

Neuroprotective outcome in clinical trials that treated head injury using hypothermia are not consistent so that deters clinicians from implementing cooling as a standard treatment. However, recent studies demonstrated that induced hypothermia has a powerful neuroprotective in transgenic mouse model of AD through up‐regulating RBM3 expression. The mild hypothermia induces significantly RBM3 expression, and RBM3 has been implicated in protection against cell death in vitro models of cooling.^30^, ^32^, ^46^ Therefore, we considered whether HT to attenuate TBI‐induced cognitive deficits and AD‐like tau phosphorylation is also mediated by RBM3 in this study.

There are lesser life‐threatening side effects in mild to moderate hypothermia (32‐34°C) treatment than in that of deeper hypothermia treatment, such as cardiac and transplant surgery, but mild to moderate hypothermia could induce RBM3 for a short period (16‐72 hours). Our preliminary experiment found that normal mice can undergo deep hypothermic treatment and refrain from life‐threatening side effects. We want to explore chronic neuroprotective role of RBM3, so we choose mouse model of deep hypothermic treatment, in which there is a durable high expression of RBM3 (Figure S1). To determine whether RBM3 involves HT‐induced neuroprotective role, the mice were performed hypothermia administration twice in two weeks and then were exposed to TBI. The data showed that HT obviously enhances RBM3 expression in mice whatever TBI treatment or not, which is consist with Bastide's study.^47^ The enhanced RBM3 expression is still maintained 4‐5 weeks after HT; meanwhile, the cognitive deficits, loss of spines and tau phosphorylation at part sites are efficaciously reversed in TBI + HT group compared with TBI group, suggesting that the enhanced RBM3 induced by cooling parallels with reduction in TBI‐induced brain injuries.

To examine the relationship between cold stress, RBM3 induction and putative neuroprotective role for TBI, we investigated whether blocking RBM3 expression with shRNA impacts neuroprotective role of HT in TBI mice pre‐treated by hypothermia. Our data demonstrated that shRNA‐RBM3 blocks HT‐induced RBM3 expression and the protective role of HT also is inhibited or abolished including cognitive deficits, loss of spines and tau phosphorylation in mouse models of TBI combined hypothermia pre‐treatment, suggesting that increased RBM3 expression is required for HT‐induced neuroprotective role for TBI mice. Our study confirms previous opinion about therapeutic hypothermia play a powerful neuroprotectant in vivo again. Moreover, we first verify that hypothermia pre‐treatment effectively attenuates cognitive and LTP deficits, loss of spines and tau phosphorylation through increased RBM3 expression in TBI mice.

RBM3 plays a crucial role in mediating synaptic plasticity essential for neuroprotection in mouse models of neurodegenerative disease.^34^, ^48^ Inducing endogenous RBM3 expression by hypothermia or overexpression by AAV mediation in vivo improves memory deficits and loss of synapses in prion and Alzheimer‐type mice, suggesting that RBM3 may be a potential target for preventing neurodegeneration disease including Alzheimer disease. Therefore, we speculated that RBM3 overexpression by AAV mediation should mimic neuroprotective role of cooling stress in TBI‐associated AD mouse models, if enhanced RBM3 expression induced by hypothermia mediates protecting against memory and LTP deficits, loss of spine and tau phosphorylation in TBI‐associated AD mouse models. Our data showed that RBM3 is effectively overexpressed in CA3 area of hippocampus by AAV‐mediated RMB3 plasmid and the deficits of pathologies and behaviours caused by TBI are reversed in TBI + AAV‐RBM3 group compared with TBI + AAV‐control, suggesting that RBM3 protects brain from memory deficits, loss of spines and tau phosphorylation at part sites in TBI mouse models.

Tau is a significant cytoskeletal protein and plays crucial roles in microtubule stability, axonal transport and neuronal function.^8^ It is not only a pathological marker of AD, but also an important pathological (physiological) feature of many neurodegenerative diseases. Our study and previous report verified that progressive neurodegeneration induced by TBI is thought to be models of tau protein‐related lesion in rodents.^6^, ^39^, ^49^ As we all know, tau phosphorylation is determined by balance of protein kinases and phosphatases, but the mechanisms of tau hyperphosphorylation still maintain elusive in tau related neurodegeneration diseases including Alzheimer disease. In the current study, we observed that hypothermia pre‐treatment reverses all sites of tau hyperphosphorylation caused by TBI including pT231, pS396, the reversed tau phosphorylation at pT231, pS396 sites is enhanced again when blocking RBM3 expression under condition of hypothermia pre‐treatment, as compared to TBI‐HT‐AVV‐control group. In other hand, overexpression RBM3 also reverses TBI‐induced tau hyperphosphorylation at pT231, pS396 and AT8 sites in mice. Taken together, these data suggested that hypothermia pre‐treatment improving AD‐like tau hyperphosphorylation is mediated by RBM3 at least in part. The mechanism of RBM3 regulating tau phosphorylation needs to be further explored in future.

Taken together, our study first demonstrated that hypothermia pre‐treatment can prevent TBI‐induced chronic AD‐like pathology and behaviour deficits through up‐regulating RBM3 expression. RBM3 may be a potential target for neurodegeneration diseases.

## 5 CONCLUSION

The hypothermia pre‐treatment may have preventative neuroprotective effect for TBI mice, as demonstrated by reversed memory and LTP deficits, loss of spines and AD‐like tau phosphorylation via RBM3. The hypothermia may be proved a new tool for risk people of suffered brain injury.

## CONFLICT OF INTEREST

The authors declare that they have no conflict of interest.

## AUTHOR CONTRIBUTION

**Bingjin Liu:** Conceptualization (equal); Data curation (lead); Formal analysis (lead); Investigation (lead); Methodology (equal); Project administration (lead); Software (lead); Validation (lead); Visualization (lead); Writing‐original draft (lead); Writing‐review & editing (lead). **Yun Cao:** Conceptualization (equal); Data curation (lead); Formal analysis (lead); Investigation (lead); Methodology (equal); Project administration (lead); Software (lead); Validation (lead); Visualization (lead); Writing‐original draft (lead); Writing‐review & editing (lead). **Fangxiao Shi:** Conceptualization (supporting); Formal analysis (supporting); Investigation (supporting); Project administration (equal); Software (equal); Validation (equal). **Lin Wang:** Data curation (supporting); Methodology (supporting); Software (equal); Validation (supporting). **Na Li:** Methodology (supporting); Software (supporting); Visualization (supporting). **Xiangshu Cheng:** Formal analysis (supporting); Methodology (supporting); Project administration (supporting). **Jin Du:** Methodology (supporting); Visualization (supporting). **Qing Tian:** Conceptualization (equal); Funding acquisition (equal); Project administration (equal); Resources (equal); Supervision (equal); Writing‐original draft (equal); Writing‐review & editing (equal). **Xinwen Zhou:** Conceptualization (lead); Funding acquisition (lead); Project administration (lead); Resources (lead); Supervision (lead); Writing‐original draft (lead); Writing‐review & editing (lead).

## Supporting information

Fig S1

## Data Availability

Data can be accessed by emailing the corresponding authors.
