# Supplementary figures and images for: The overexpression of RBM3 alleviates TBI‐induced behaviour impairment and AD‐like tauopathy in mice
Source: J Cell Mol Med. 2020 Jul 10;24(16):9176–88. doi: 10.1111/jcmm.15555 (PMC7417709; doi:10.1111/jcmm.15555)

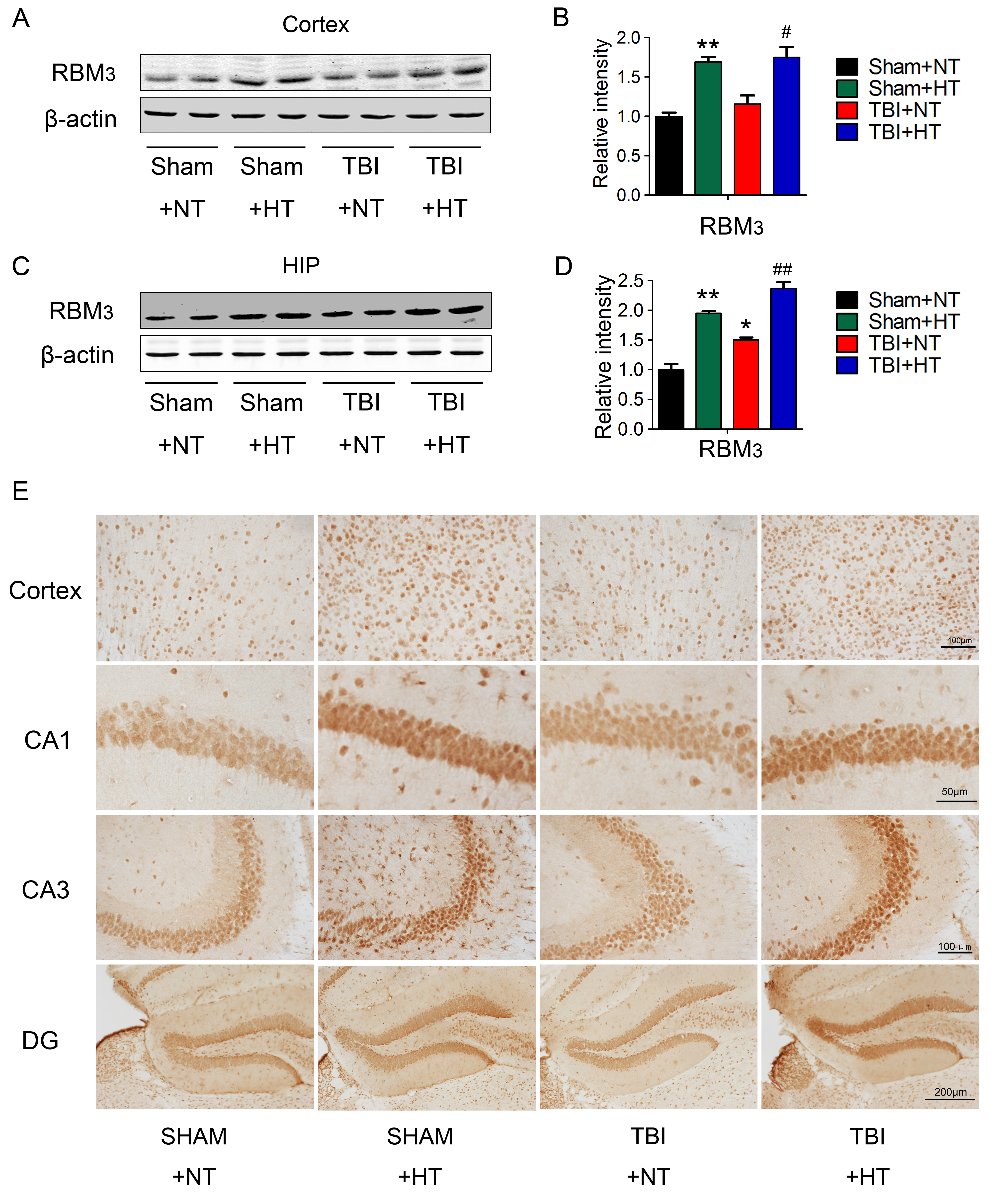

Supplement: Supplementary file 1 — Fig S1 [file JCMM-24-9176-s001.jpg]
